# Hsa-let-7c-5p augments enterovirus 71 replication through viral subversion of cell signaling in rhabdomyosarcoma cells

**DOI:** 10.1186/s13578-017-0135-9

**Published:** 2017-01-14

**Authors:** Bingfei Zhou, Min Chu, Shanshan Xu, Xiong Chen, Yongjuan Liu, Zhihao Wang, Fengfeng Zhang, Song Han, Jun Yin, Biwen Peng, Xiaohua He, Wanhong Liu

**Affiliations:** 1 Hubei Province Key Laboratory of Allergy and Immunology, School of Basic Medical Sciences, Wuhan University, No. 185, Donghu Road, Wuchang District, Wuhan, 430071 China; 2 Hubei Provincial Key Laboratory of Developmentally Originated Disease, School of Basic Medical Sciences, Wuhan University, Wuhan, 430071 China

**Keywords:** Hsa-let-7c-5p, EV71, MAP4K4, JNK

## Abstract

**Background:**

Human enterovirus 71 (EV71) causes severe hand, foot and mouse disease, accompanied by neurological complications. During the interaction between EV71 and the host, the virus subverts host cell machinery for its own replication. However, the roles of microRNAs (miRNAs) in this process remain obscure.

**Results:**

In this study, we found that the miRNA hsa-let-7c-5p was significantly upregulated in EV71-infected rhabdomyosarcoma cells. The overexpression of hsa-let-7c-5p promoted replication of the virus, and the hsa-let-7c-5p inhibitor suppressed viral replication. Furthermore, hsa-let-7c-5p targeted mitogen-activated protein kinase kinase kinase kinase 4 (MAP4K4) and inhibited its expression. Interestingly, downregulation of MAP4K4 expression led to an increase in EV71 replication. In addition, MAP4K4 knockdown or transfection with the hsa-let-7c-5p mimic led to activation of the c-Jun NH2-terminal kinase (JNK) signaling pathway, whereas the hsa-let-7c-5p inhibitor inhibited activation of this pathway. Moreover, EV71 infection promoted JNK pathway activation to facilitate viral replication.

**Conclusions:**

Our data suggested that hsa-let-7c-5p facilitated EV71 replication by inhibiting MAP4K4 expression, which might be related to subversion of the JNK pathway by the virus. These results may shed light on a novel mechanism underlying the defense of EV71 against cellular responses. In addition, these findings may facilitate the development of new antiviral strategies for use in future therapies.

**Electronic supplementary material:**

The online version of this article (doi:10.1186/s13578-017-0135-9) contains supplementary material, which is available to authorized users.

## Background

Human enterovirus 71 (EV71), which belongs to the genus *Enterovirus* in the family *Picornaviridae*, is a type of single-stranded positive-sense RNA virus that is known to be a major cause of hand, foot and mouth disease (HFMD) in young children. This virus may cause serious neurological complications compared with other pathogens associated with HFMD [1]. Since EV71 was first isolated in 1969 [2], several serious infectious outbreaks have occurred, placing heavy burdens on society [3–7]. However, no effective drug has been identified for clinical application. Therefore, EV71 infection remains a public health concern.

MicroRNAs (miRNAs), a class of highly conserved small noncoding-RNAs, are 18–25 nucleotides (nts) in length. They post-transcriptionally modulate gene expression via mRNA degradation, mRNA cleavage, and translational repression [8–10]. MiRNAs are involved in a number of biological processes, such as cell cycle progression, cell survival and apoptosis [11–14]. Increasing evidence indicates that miRNAs also play a role in the pathogenesis of viral infectious diseases. Both DNA and RNA viruses can act as boosters, destroyers and hijackers in the regulation of cellular miRNAs to facilitate progression of their life cycles [15, 16].

The lethal-7 (let-7) miRNA was first discovered in *Caenorhabditis elegans* (*C. elegans*), in which it acts as a key developmental regulator [17]. The *Homo sapiens* (*H. sapiens*) let-7 (hsa-let-7) family contains 13 individual members encoded on different chromosomes. The expression of these miRNAs, except for that of hsa-let-7c, is meticulously regulated by lin28 [18–20]. Hsa-let-7c is downregulated in most cancers and plays crucial roles in carcinogenesis and cancer metastasis [21–23]. In addition, it has been reported to be upregulated in response to RNA virus infection and to affect viral replication [24–26]. The miRNA let-7c has been shown to inhibit influenza virus replication by degrading viral gene M1 (+) cRNA in human lung epithelial cells [24]. Further, let-7c has been reported to suppress viral replication through targeting of the transcription factor BACH1 in dengue virus-infected human hepatoma Huh-7 cells [25]. Moreover, this miRNA may enhance HIV-1 infection by downregulating the cellular restriction factor p21 [26]. However, its roles in mediating virus-host interactions through the regulation of host pathways have not been previously investigated. In addition, the regulation of hsa-let-7c-5p in EV71 replication has not yet been reported.

In this study, we showed that the hsa-let-7c-5p-mediated regulation of mitogen-activated protein kinase kinase kinase kinase 4 (MAP4K4) might play an important role in EV71 replication. Hsa-let-7c-5p may modulate viral subversion of the c-Jun NH2-terminal kinase (JNK) signaling pathway and promote EV71 replication.

## Results

### Mature miRNA hsa-let-7c-5p expression is upregulated during EV71 infection

**Fig. 1:**
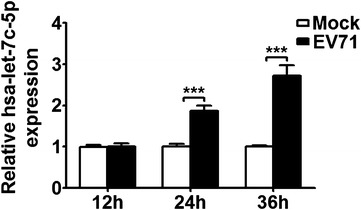
Relative expression of mature hsa-let-7c-5p is regulated by EV71 infection. RD cells were infected with EV71 (MOI = 5) or not (mock infection) at the indicated time points. The expression of hsa-let-7c-5p was detected by stem-loop qRT-PCR and normalized to that of U6 snRNA. The data are presented as the mean ± SD from one representative experiment, and all results here and in all subsequent figures were replicated three times. Significant differences are indicated as follows: ***p < 0.001

According to current and our previous studies, rhabdomyosarcoma (RD) cells and the Henrietta Lacks strain of cancer (HeLa) cells can be used as in vitro models to investigate the interplay between EV71 and its host [16, 27–29]. To determine whether hsa-let-7c-5p is involved in EV71 infection, we first examined the expression of mature hsa-let-7c-5p in uninfected and EV71-infected RD cells by stem-loop quantitative reverse transcription polymerase chain reaction (qRT-PCR). The effect of EV71 infection on hsa-let-7c-5p expression was detected at 12, 24 and 36 h post-infection (p.i.), and the results showed that hsa-let-7c-5p expression was significantly increased in EV71-infected RD cells compared with mock-infected cells at 24 h p.i. (1.9-fold change) and 36 h p.i. (2.7-fold change) (Fig. 1, *p* < 0.001). Similar hsa-let-7c-5p expression was detected in EV71-infected HeLa cells (Additional file 1: Figure S1). These data demonstrated that EV71 infection upregulated the expression of hsa-let-7c-5p.

### The overexpression of hsa-let-7c-5p promotes EV71 replication

**Fig. 2:**
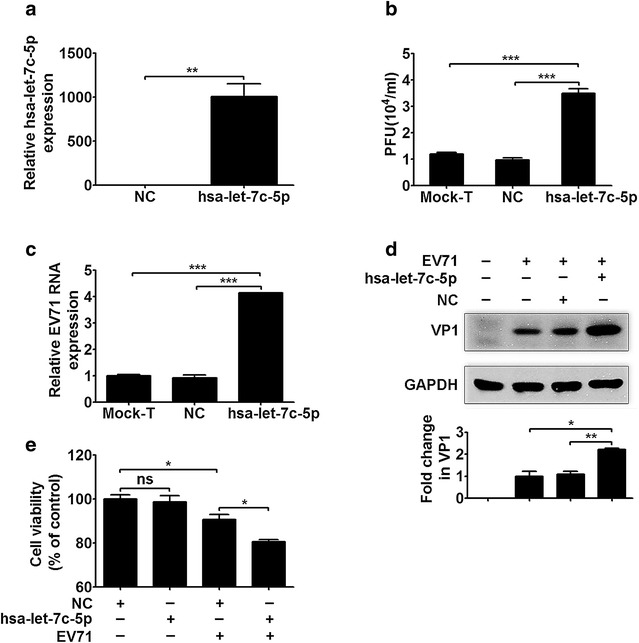
EV71 replication is regulated by the hsa-let-7c-5p mimic. **a** The efficiency of transfection of the hsa-let-7c-5p mimic (100 nM) into RD cells for 48 h was determined by stem-loop qRT-PCR. **b** The EV71 titer is altered by the hsa-let-7c-5p mimic. RD cells were transfected with the NC (100 nM), hsa-let-7c-5p (100 nM) or neither (mock transfection, Mock-T) for 48 h, followed by EV71 infection (MOI = 5) for 24 h. Total virus was collected from infected cells and culture supernatants and used in plaque assays. The virus titers in the cultures are expressed as plaque-forming units (PFU) per milliliter. **c** The expression of viral RNA and (**d**) the viral structural protein VP1 was assessed by qRT-PCR and Western blotting, respectively, after transfection of cells with the hsa-let-7c-5p mimic, and GAPDH was used as an internal control. RD cells were transfected with the NC (100 nM), hsa-let-7c-5p (100 nM) or neither for 48 h, followed by EV71 infection (MOI = 5) for 24 h. In addition, RD cells were subjected to mock infection, as shown in (**d**), to assess specificity of the VP1 primary antibody. The *lower panel* in (**d**) shows that VP1 expression normalized to GAPDH is expressed as a fold change compared with the Mock-T group, as quantified using Image J software. **e** The effect of the hsa-let-7c-5p mimic on cell viability was assessed by MTT assay. RD cells were transfected with the NC (100 nM) or hsa-let-7c-5p mimic (100 nM) for 48 h, followed by EV71 infection (MOI = 5) or mock infection for 24 h. Cell viability is expressed as percentages of the NC-transfected and mock-infected cells, which were used as controls. *p < 0.05; **p < 0.01; ***p < 0.001; *ns* no significance

To investigate the role of hsa-let-7c-5p during EV71 infection, we first assessed the transfection efficiency of an hsa-let-7c-5p miRNA mimic in RD cells. A negative control (NC) miRNA mimic was also used in these experiments. Markedly increased hsa-let-7c-5p expression (by nearly 1000-fold) was observed in the hsa-let-7c-5p-overexpressing group compared with the NC group (Fig. 2a, *p* < 0.01). As shown in Additional file 1: Figure S2 and Fig. 2b, the hsa-let-7c-5p mimic at concentrations of 50 nM (*p* < 0.01), 70 nM (*p* < 0.001) and 100 nM (*p* < 0.001) increased the virus titer compared with the NC mimic, and the highest virus titer was detected following treatment with 100 nM of the hsa-let-7c-5p mimic. Thus, 100 nM of the hsa-let-7c-5p mimic was used in the following experiments. A substantially increased virus titer was observed in the hsa-let-7c-5p mimic group compared with the mock transfection (Mock-T) group (Fig. 2b, *p* < 0.001). Further, the levels of viral RNA and the structural protein VP1 were significantly increased in the hsa-let-7c-5p mimic group compared with the NC mimic and Mock-T groups (Fig. 2c, *p* < 0.001; Fig. 2d, *p* < 0.01 and *p* < 0.05, respectively). No difference in the virus titer, viral RNA level or VP1 protein level was observed between the NC mimic and Mock-T groups (Fig. 2b–d). Next, to exclude the possibility that hsa-let-7c-5p affects host cells, cell viability was assessed by 3-(4,5-dimethylthiazol-2-yl)-2,5-diphenyltetrazolium bromide (MTT) assay. No significant difference in cell viability was observed between hsa-let-7c-5p mimic- and NC mimic-transfected mock-infected RD cells (Fig. 2e). However, the hsa-let-7c-5p mimic reduced the survival of EV71-infected RD cells in contrast with the NC mimic (Fig. 2e, *p* < 0.05). Taken together, these data demonstrated that an increased hsa-let-7c-5p level might promote EV71 replication and facilitate the viral impairment of host cells.

### The inhibition of hsa-let-7c-5p expression represses EV71 replication

To further confirm the effect of hsa-let-7c-5p on EV71 replication, cells were treated with a miRNA inhibitor. This inhibitor, referred to as hsa-let-7c-5pi in the paper, is a synthetic oligonucleotide with a sequence that is exactly complementary to hsa-let-7c-5p. A negative control miRNA inhibitor (NCi) was also used, which is a miRNA oligonucleotide from *C. elegans* whose sequence shows almost no homology with the genomes of *H. sapiens*, *Rattus norvegicus* and *Mus musculus*.

**Fig. 3:**
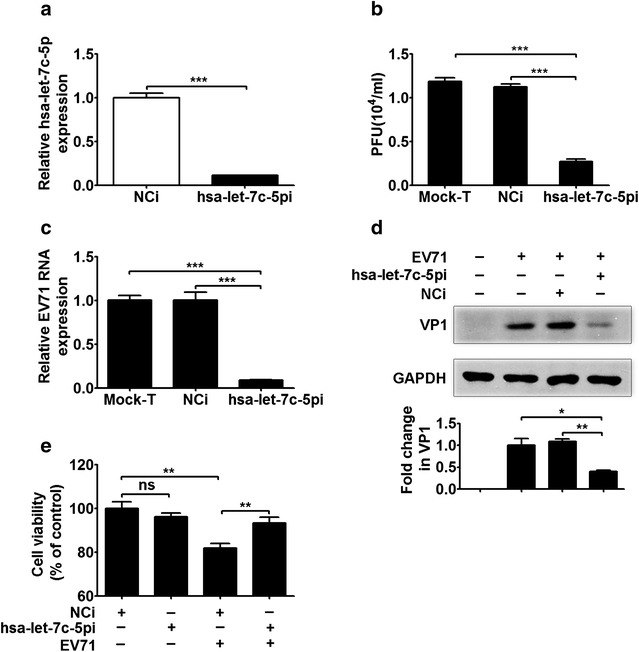
EV71 replication is modulated by the hsa-let-7c-5p inhibitor. **a** The effect of transfection of hsa-let-7c-5pi (150 nM) into RD cells for 48 h on hsa-let-7c-5p expression was assessed by stem-loop qRT-PCR. **b** The virus titer is regulated by hsa-let-7c-5pi. RD cells were transfected with NCi (150 nM), hsa-let-7c-5pi (150 nM) or neither for 48 h and then infected with EV71 (MOI = 5) for 24 h. Total virus was collected from infected cells and culture supernatants and used in plaque assays. The virus titers in the cultures are expressed as PFU per milliliter. **c** Effects of the hsa-let-7c-5p mimic on the expression of viral RNA and (**d**) the viral structural protein VP1 were analyzed by qRT-PCR and Western blotting, respectively, using GAPDH as an internal control. RD cells were transfected with NCi (150 nM), hsa-let-7c-5pi (150 nM) or neither for 48 h, followed by EV71 infection (MOI = 5) for 24 h. The RD cells were subjected to mock infection, as shown in (**d**), to assess the specificity of the VP1 primary antibody. The *lower panel* in (**d**) shows that VP1 expression normalized to GAPDH is expressed as a fold change compared with the Mock-T group, as quantified using Image J software. **e** The effect of hsa-let-7c-5pi on cell viability was assessed by MTT assay. RD cells were transfected with NCi (150 nM) or hsa-let-7c-5pi (150 nM) for 48 h, followed by infection with or without EV71 (MOI = 5) for 24 h. Cell viability is expressed as percentages of the NCi-transfected and mock-infected cells, which were used as controls. *p < 0.05; **p < 0.01; ***p < 0.001; *ns* no significance

To evaluate the effect of hsa-let-7c-5pi on hsa-let-7c-5p expression, 150 nM inhibitor oligonucleotide was transfected into RD cells. Stem-loop qRT-PCR revealed that hsa-let-7c-5p expression was significantly decreased in the hsa-let-7c-5pi group compared with the NCi group (Fig. 3a, *p* < 0.001). In addition, the plaque assay results showed that hsa-let-7c-5pi significantly reduced the EV71 titer of the hsa-let-7c-5pi group compared with the NCi and Mock-T groups (Fig. 3b, *p* < 0.001). Further, both the viral RNA and VP1 protein levels were significantly suppressed in the hsa-let–5pi group compared with the NCi and Mock-T groups, as shown in Fig. 3c (*p* < 0.001) and Fig. 3d (*p* < 0.01 and *p* < 0.05, respectively). Moreover, no significant differences in the virus titer, viral RNA level or VP1 protein level were detected between the NCi and Mock-T groups (Fig. 3b–d). MTT assays revealed no significant difference in cell viability between the mock-infected RD cells transfected with hsa-let-7c-5pi and those transfected with NCi (Fig. 3e). However, viability of the EV71-infected RD cells was enhanced in the hsa-let-7c-5pi group compared with the NCi group (Fig. 3e, *p* < 0.01). These results showed that the hsa-let-7c-5p inhibitor suppressed EV71 replication and protected host cells from EV71-induced cell death.

### Hsa-let-7c-5p directly inhibits the expression of MAP4K4 by binding to its 3′UTR

To characterize the molecular mechanism associated with hsa-let-7c-5p function, we used an online miRNA prediction program, TargetScan, and the Starbase database to comprehensively analyze potential targets [30–32]. The results showed that MAP4K4 was a potential candidate target of hsa-let-7c-5p.

**Fig. 4:**
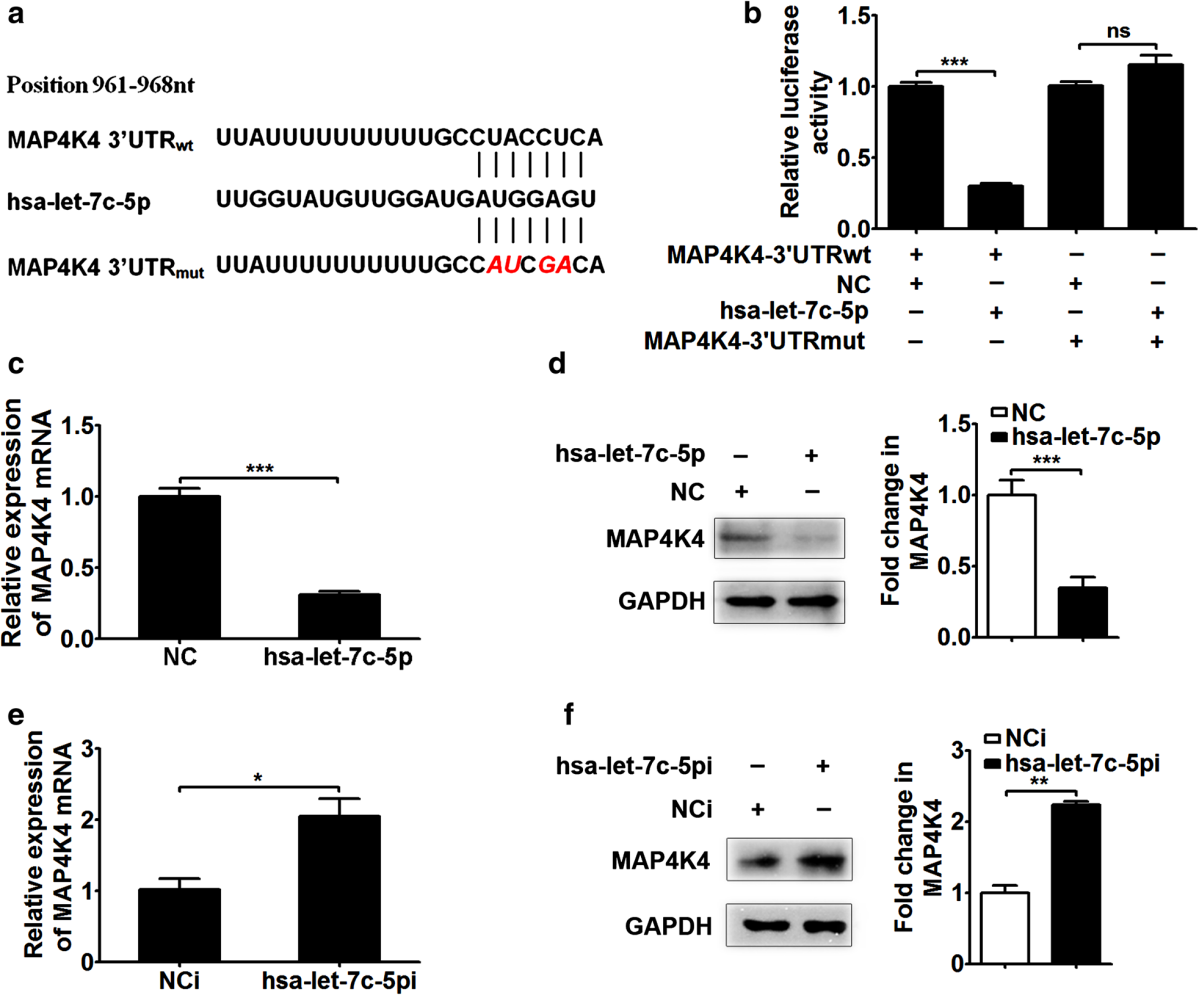
Target of hsa-let-7c-5p in MAP4K4. **a** Predicted binding site of hsa-let-7c-5p to the 3′UTR of human MAP4K4, as determined using TargetScan. **b** MAP4K4 was validated as a target of hsa-let-7c-5p by dual luciferase reporter assay. RD cells were transfected with a wild-type or mutant 3′UTR vector (50 ng) for 6 h, followed by transfection of the NC (100 nM) or hsa-let-7c-5p mimic (100 nM) for 48 h. *Renilla* luciferase activity in each sample was normalized to *Firefly* luciferase activity. **c** The effect of the hsa-let-7c-5p mimic on the MAP4K4 mRNA level. RD cells were transfected with NC (100 nM) or hsa-let-7c-5p (100 nM) for 48 h. RNA was extracted and analyzed by qRT-PCR. **d** Regulation of the MAP4K4 protein level by the transfection of RD cells with the hsa-let-7c-5p mimic (100 nM) for 48 h, as demonstrated by Western blotting. The *right panel* in (**d**) shows that MAP4K4 expression normalized to GAPDH is expressed as a fold change compared with the NC mimic group, as quantified using Image J software. **e** The MAP4K4 mRNA level is affected by hsa-let-7c-5pi. RD cells were separately transfected with NCi (150 nM) and hsa-let-7c-5pi (150 nM) for 48 h, and then RNA was analyzed by qRT-PCR. **f** Regulation of the MAP4K4 protein level by the transfection of RD cells with hsa-let-7c-5pi (150 nM) for 48 h, as demonstrated by Western blotting. The *right panel* in (**f**) shows that MAP4K4 expression normalized to GAPDH is expressed as a fold change compared with the NCi group, as quantified using Image J software. *p < 0.05; **p < 0.01; ***p < 0.001; *ns* no significance

To date, the standard strategy used to validate miRNA targets involves artificial sensor assay, in which the 3′UTR of a gene of interest is coupled to a reporter plasmid. A schematic diagram of the binding site of hsa-let-7c-5p to the 3′UTR of the MAP4K4 mRNA is presented in Fig. 4a, and mutations in the sequence are highlighted in red and italic font. The luciferase assay results showed that the reporter activity of psi-MAP4K4-3′UTR was significantly decreased in the hsa-let-7c-5p mimic group compared with the NC group (Fig. 4b, *p* < 0.001), whereas that of psi-MAP4K4-3′UTRmut did not significantly differ between the two groups (Fig. 4b).

To investigate the post-transcriptional regulation of hsa-let-7c-5p, we detected the MAP4K4 mRNA and protein levels by qRT-PCR and Western blotting, respectively. The hsa-let-7c-5p mimic at a concentration of 100 nM significantly reduced MAP4K4 mRNA expression compared with the NC mimic (Fig. 4c, *p* < 0.001). In addition, the MAP4K4 protein level was obviously decreased in the hsa-let-7c-5p mimic group compared with the NC group (Fig. 4d, *p* < 0.001). To further validate the miRNA-target interaction, cells were treated with an hsa-let-7c-5p inhibitor to assess the regulatory effects of this miRNA at both the mRNA and protein levels. The hsa-let-7c-5p inhibitor oligonucleotide at a concentration of 150 nM increased the MAP4K4 mRNA and protein levels compared with the NC inhibitor (Fig. 4e, *p* < 0.05; Fig. 4f, *p* < 0.01). These data demonstrated that MAP4K4 was a target of hsa-let-7c-5p and that hsa-let-7c-5p post-transcriptionally regulated MAP4K4 expression by directly binding to its 3′UTR.

### MAP4K4 knockdown facilitates EV71 replication and activates the JNK signaling pathway

**Fig. 5:**
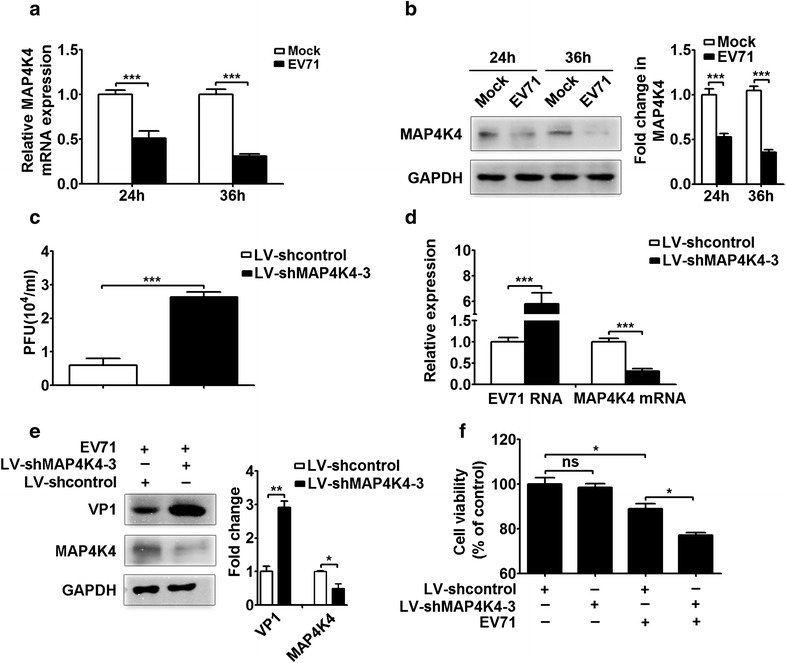
The effect of MAP4K4 knockdown on EV71 replication. **a** qRT-PCR and, **b** Western blotting were performed to assess the expression of MAP4K4 in response to EV71 infection in RD cells. RD cells were infected with or without EV71 at 5 MOI at the indicated time points. The *right panel* in (**b**) shows that the MAP4K4 protein level normalized to GAPDH is expressed as a fold change compared with the mock infection (24 h) group, as quantified using Image J software. **c** The effect of MAP4K4 knockdown on the virus titer was determined at 24 h p.i. by plaque assays. RD cells were transfected with the LV-shcontrol or LV-shMAP4K4-3 vector for 48 h, followed by infection with EV71 (MOI = 5) for 24 h. Total virus was collected from infected cells and culture supernatants and used in plaque assays. The virus titers in the cultures are expressed as PFU per milliliter. **d** Viral RNA and MAP4K4 mRNA expression and (**e**) VP1 protein and MAP4K4 protein expression following MAP4K4 knockdown were determined by qRT-PCR and Western blotting, respectively. The LV-shcontrol or LV-shMAP4K4-3 vector was transfected into RD cells for 48 h, followed by EV71 infection (MOI = 5) for 24 h. The *right panel* in (**e**) shows that the VP1 and MAP4K4 expression levels normalized to GAPDH are expressed as fold changes compared with the LV-shcontrol-transfected group, as quantified using Image J software. **f** The effect of MAP4K4 silencing on cell viability was assessed by MTT assay. RD cells were transfected with the LV-shcontrol (140 ng) or LV-shMAP4K4-3 (140 ng) vector for 48 h and then infected with or without EV71 (MOI = 5) for 24 h. Cell viability is expressed as percentages of the LV-shcontrol-transfected and mock-infected cells, which were used as controls. *p < 0.05; **p < 0.01; ***p < 0.001; *ns* no significance

To detect whether MAP4K4 is involved in EV71 infection, we performed qRT-PCR and Western blotting. The results showed that MAP4K4 expression was significantly decreased at both the mRNA and protein levels in EV71-infected RD cells compared with mock-infected cells at 24 and 36 h p.i. (Fig. 5a, b; *p* < 0.001). To further determine whether MAP4K4 affects EV71 replication, an shRNA pool was used to silence MAP4K4 expression. MAP4K4 expression was effectively silenced at both the mRNA and protein levels by the LV-shMAP4K4 vector in RD cells (Additional file 1: Figure S3). In addition, MAP4K4 expression was more strongly repressed by the LV-shMAP4K4-3 vector than by the other two vectors (Additional file 1: Figure S3). Thus, the LV-shMAP4K4-3 vector was used in the subsequent experiments. A significantly increased EV71 titer was detected in the LV-shMAP4K4-3-transfected group compared with the LV-shcontrol-transfected group (Fig. 5c, *p* < 0.001). Moreover, EV71-infected RD cells transfected with the LV-shMAP4K4-3 vector exhibited significantly reduced MAP4K4 expression at both the mRNA and protein levels compared with those transfected with the LV-shcontrol vector (Fig. 5d, *p* < 0.001; Fig. 5e, *p* < 0.05). Further, the EV71 RNA and VP1 protein levels were significantly enhanced in the LV-shMAP4K4-3-transfected RD cells compared with the LV-shcontrol-transfected cells (Fig. 5d, *p* < 0.001; Fig. 5e, *p* < 0.01). The MTT assay results showed no significant difference in cell viability between normal RD cells transfected with the LV-shMAP4K4-3 vector and those transfected with the LV-shcontrol vector (Fig. 5f). However, the viability of EV71-infected RD cells was significantly decreased in the LV-shMAP4K4-3-tranfected group compared with the LV-shcontrol-transfected group (Fig. 5f, *p* < 0.05). Thus, we concluded that EV71 infection resulted in the downregulation of MAP4K4 expression and that knockdown of MAP4K4 promoted EV71 replication.

**Fig. 6:**
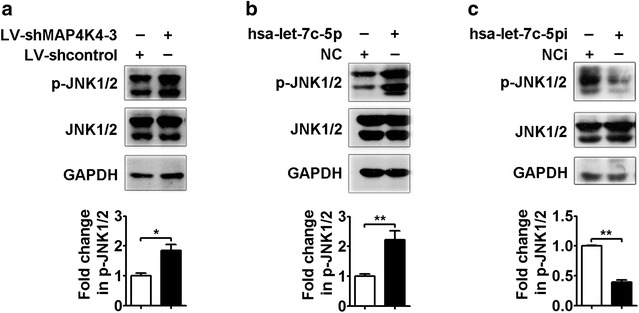
Regulation of the JNK signaling pathway by MAP4K4 and hsa-let-7c-5p. **a** The LV-shMAP4K4-3 vector, **b** hsa-let-7c-5p mimic (100 nM), **c** hsa-let-7c-5pi (150 nM) or corresponding control was transfected into RD cells for 48 h. The total JNK1/2 and p-JNK1/2 protein levels were analyzed by Western blotting. The ratios of the p-JNK1/2 to total JNK1/2 signals are expressed as fold changes compared with the corresponding controls, as quantified using Image J software (*lower panels*). *p < 0.05; **p < 0.01

To further investigate the regulatory mechanism of EV71 replication by MAP4K4, the expression of proteins involved in the two major signaling pathways downstream of MAP4K4 (the JNK and NF-κB pathways) was assessed by Western blotting. The results showed that the ratio of the phosphorylated JNK1/2 (p-JNK1/2) protein level to the total JNK1/2 protein level was higher in the LV-shMAP4K4-3-transfected group than in the LV-shcontrol-transfected group (Fig. 6a, *p* < 0.05). The hsa-let-7c-5p mimic similarly increased the p-JNK1/2 protein level (Fig. 6b, *p* < 0.01). In contrast, the p-JNK1/2 protein level was significantly reduced in the hsa-let-7c-5pi group compared with the NCi group (Fig. 6c, *p* < 0.01). However, the phosphorylated NF-κB p65 (p-NF-κB p65) protein level was not significantly differ between RD cells transfected with the LV-shMAP4K4-3 vector and those with the LV-shcontrol vector (Additional file 1: Figure S3). Similar results were observed in the hsa-let-7c-5p mimic and hsa-let-7c-5pi groups compared with their corresponding control groups (Additional file 1: Figure S3). These data showed that MAP4K4 knockdown and the hsa-let-7c-5p mimic activated the JNK signaling pathway but not the NF-κB pathway, whereas JNK pathway activation was suppressed by the hsa-let-7c-5p inhibitor.

### Subversion of the JNK signaling pathway by EV71 promotes viral replication

**Fig. 7:**
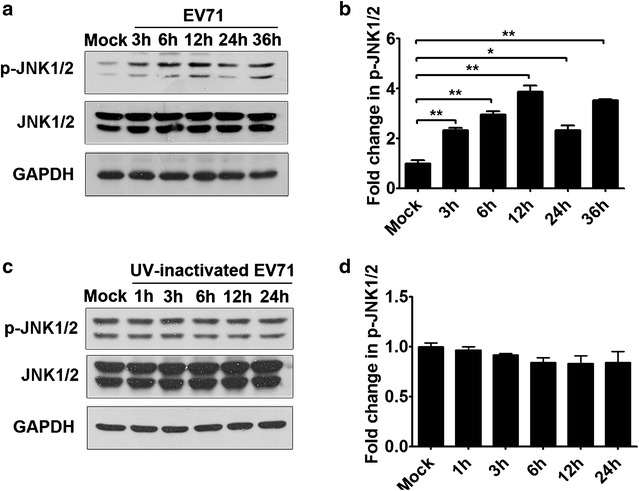
Modulation of JNK pathway activity by EV71 infection. **a** The effect of EV71 infection on JNK activity was assessed by Western blotting. RD cells were infected with EV71 (MOI = 5) at the indicated time points, and the data were analyzed by comparison with the mock-infected group. The ratios of the p-JNK1/2 to total JNK1/2 signals are expressed as fold changes compared with the mock-infected group, as quantified using Image J software, as shown in (**b**). **c** The effect of UV inactivation of EV71 on JNK activity was assessed by Western blotting. RD cells were infected with UV-inactivated EV71 at the indicated time points, and the data were analyzed by comparison with the mock-infected group. The ratios of the p-JNK1/2 to total JNK1/2 signals are expressed as fold changes compared with the mock-infected group, as quantified using Image J software, as shown in (**d**). *p < 0.05; **p < 0.01

As demonstrated above, the JNK signaling pathway is modulated by MAP4K4 and hsa-let-7c-5p. To investigate whether this pathway is involved in EV71 replication in RD cells, we first detected JNK signaling pathway activity during EV71 infection by Western blotting. Total JNK1/2 expression remained unchanged in EV71-infected RD cells at 3, 6, 12, 24 and 36 h compared with that in mock-infected cells (Fig. 7a). However, the phosphorylated JNK1/2 level was increased in the EV71-infected cells at 3, 6, 12, 24 and 36 h compared with the mock-infected cells, with peak expression observed at 12 h (Fig. 7a, b). To determine whether JNK pathway activity is affected during the early stage of infection, cells were infected with UV-inactivated EV71. The results showed that UV-inactivated EV71 infection had no effect on the p-JNK1/2 or total JNK1/2 protein level at 1, 3, 6, 12 or 24 h p.i. compared with mock infection (Fig. 7c, d). These data suggested that EV71 replication, but not EV71 infection, activated the JNK pathway.

**Fig. 8:**
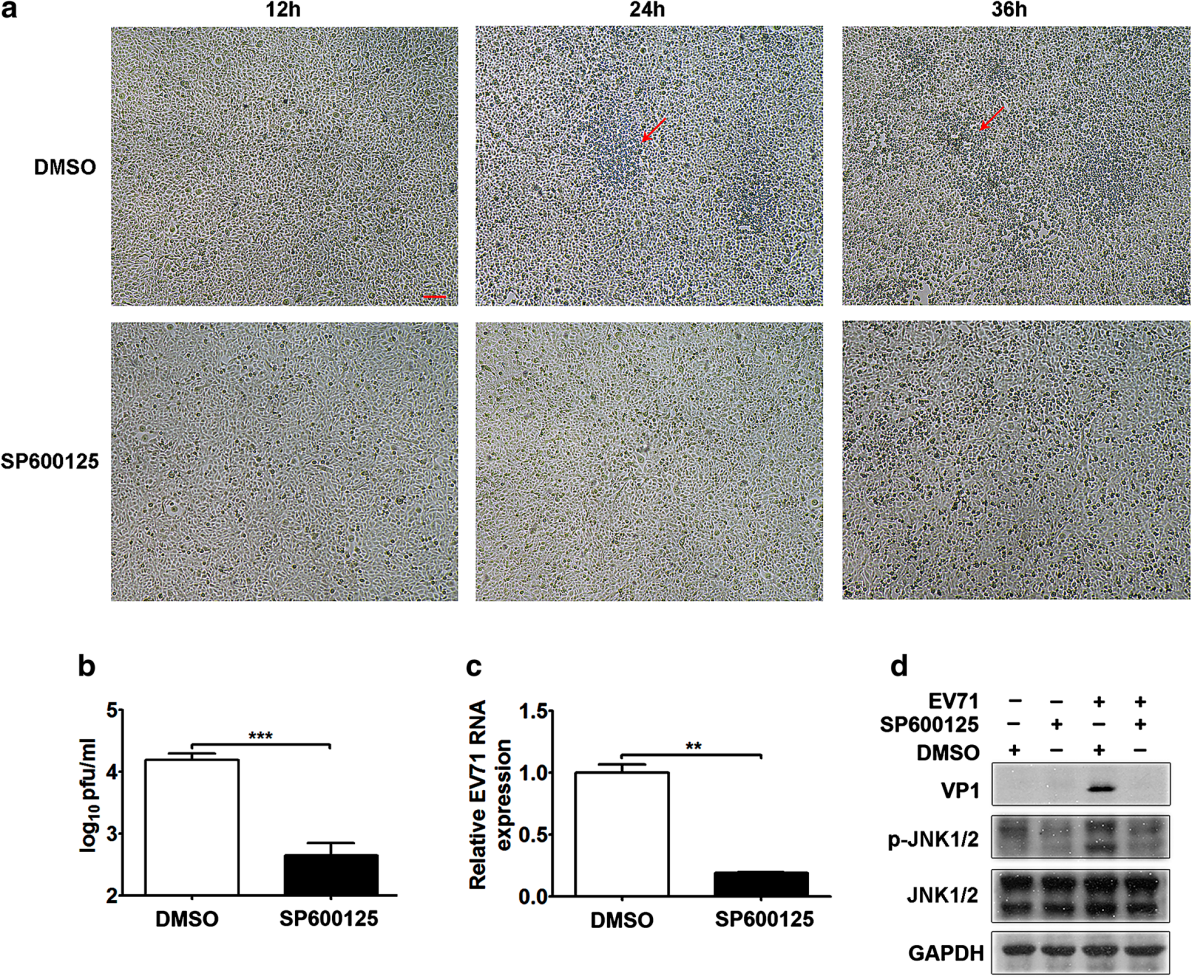
Impact of the JNK inhibitor SP600125 on EV71 replication. **a** Effect of SP600125 on the CPE of EV71-infected RD cells. RD cells were pretreated with or without SP600125 for 1 h and infected with EV71 (MOI = 5) for 12, 24 or 36 h. Cell morphology was examined by light microscopy (40×). The CPE is labeled with red arrows. The* scale bar* is 100 μm, as shown in the first image. **b** Regulation of the virus titer by SP600125 at 24 h p.i. was assessed by plaque assays. The virus titers in the cultures are expressed as PFU per milliliter. **c** Regulation of the expression of EV71 RNA by SP600125 at 24 h p.i. was examined by qRT-PCR. **d** The effects of SP600125 on the VP1, total JNK1/2 and p-JNK1/2 protein levels were assessed by Western blotting. RD cells were pretreated with or without SP600125 for 1 h prior to EV71 infection (MOI = 5) or mock infection for 24 h. **p < 0.01; ***p < 0.001

To further confirm the link between EV71 replication and the JNK pathway in RD cells, we utilized a specific inhibitor of JNK, SP600125. No obvious cytopathic effect (CPE) on RD cells was observed at 24 or 36 h p.i. in the SP600125-treated group compared with the dimethyl sulfoxide (DMSO)-treated group (Fig. 8a). Furthermore, the virus titer was significantly reduced in the SP600125-treated group compared with the DMSO-treated group (Fig. 8b, *p* < 0.001). Moreover, the EV71 RNA and VP1 protein levels were decreased by the SP600125 treatment compared with the DMSO treatment (Fig. 8c, *p* < 0.01; Fig. 8d). These data demonstrated that EV71-induced JNK pathway activation facilitated EV71 replication in RD cells.

## Discussion

Here, we reported that EV71 infection resulted in upregulation of expression of the cellular miRNA hsa-let-7c-5p and that hsa-let-7c-5p overexpression promoted viral replication by targeting MAP4K4 to activate the JNK signaling pathway in RD cells. These results suggested that EV71 might utilize hsa-let-7c-5p to subvert signaling pathways for its own benefit.

It is important to elucidate the interactions between EV71 and host cellular miRNAs. Comprehensive miRNA profiling has been performed using a deep sequencing approach, and certain miRNAs have been proposed to participate in crucial interactions with EV71 [33, 34]. On the one hand, host miRNAs may target the viral genome directly to suppress viral replication. Wen et al. [35] have reported that miRNA-23b expression is downregulated by EV71 infection and that this miRNA inhibits viral replication via suppression of the structural protein VP1. In addition, Zheng et al. [36] have reported that hsa-miR-295-5p expression is upregulated by EV71 infection and that this miRNA suppresses viral replication by targeting the VP1 and VP3 coding regions. On the other hand, host miRNAs may regulate EV71 replication by targeting host factors and immune signaling pathways [37, 38]. Tang et al. [37] have reported that host miR-197 expression is downregulated by EV71 and that this miRNA represses viral replication by targeting the host RAN, member RAS oncogene family protein. In addition, Xu et al. [38] have reported that host miR-526a expression is upregulated by EV71 and that this miRNA attenuates viral replication by suppressing CYLD expression. Previously, we have found that miR-27a expression is downregulated and that hsa-let-7c-5p expression is upregulated in RD cells during EV71 infection. Further, we have reported that miR-27a overexpression suppresses viral replication through the regulation of EGFR-mediated pathways [16]. It is of interest to investigate the role of hsa-let-7c-5p miRNA upregulation in EV71 infection. In this study, we showed that the upregulation of hsa-let-7c-5p expression by EV71 promoted viral replication by activating the JNK signaling pathway downstream of MAP4K4.

MAP4K4, a member of the human STE20/mitogen-activated protein kinase kinase kinase family of serine/threonine kinases, is involved in multiple cellular processes, such as cell motility, cytoskeletal rearrangement, and cell proliferation, as well as a complex network of signaling pathways. MAP4K4 is mainly associated with different types of cancer and diabetes [39, 40]. Notably, the roles of MAP4K4 in these biological processes and diseases involve the JNK and NF-κB signaling pathways [41–43]. MAP4K4 deficiency has been reported to stabilize TRAF2 and induce TRAF2/IL-6 upregulation, leading to insulin resistance [44]. In addition, MAP4K4 depletion has been shown to prevent primary CD4^+^ T cell proliferation and activation in vitro and to reduce the expression of IL-2 and IFN-γ [45]. Further, JNK phosphorylation has been demonstrated to be enhanced by MAP4K4 depletion in CD4^+^ T cells exposed to ozone-oxidized black carbon [46]. However, little is known regarding the function of MAP4K4 in EV71-infected RD cells. In our study, MAP4K4 knockdown also enhanced JNK1/2 phosphorylation. Moreover, EV71 infection resulted in the downregulation of MAP4K4 expression, and the silencing of MAP4K4 led to increased viral replication. These results implied that MAP4K4 might function as a novel antiviral regulator of EV71 replication.

Mammalian JNKs are encoded by three distinct genes, namely *jnk*1, *jnk*2 and *jnk*3. JNK1 and JNK2 are widely expressed in most cell types, and JNK3 expression is localized to the brain and testes [47]. JNK activation is mediated by inflammatory cytokines (such as TNF, IL-1 and TGF-β), Toll-like receptors and ligation of antigen receptors. The JNK pathway has been implicated in multiple diseases, including infectious diseases [48]. The JNK pathway has been shown to be required for the viral replication of herpes simplex virus, hepatitis B virus, hepatitis C virus, rotavirus, HIV-1 and human cytomegalovirus [49–54]. In addition, EV71 infection has been reported to activate the JNK pathway in immature dendritic cells and to promote the production of inflammatory cytokines, such as IL-6, IL-10 and TNF-α, whereas inhibition of this pathway results in the suppression of viral replication and the reduced secretion of cytokines [55]. In our study, EV71 replication activated the JNK pathway in RD cells, and blocking of the JNK pathway led to the inhibition of viral replication.

## Conclusions

Collectively, the results of the present study have demonstrated that the hsa-let-7c-5p inhibitor and MAP4K4 might act as anti-EV71 modulators. Hsa-let-7c-5p might promote EV71 replication by targeting MAP4K4 expression. Moreover, hsa-let-7c-5p facilitates EV71 replication partly through the MAP4K4/JNK axis. EV71 infection may induce the upregulation of hsa-let-7c-5p, along with the downregulation of MAP4K4 and activation of the JNK pathway. These findings may indicate that EV71 uses an escape mechanism involving hsa-let-7c-5p-mediated regulation of the MAP4K4/JNK pathway to promote its own replication.

## Methods

### Cell culture, transfection and reagents

RD cells and African green monkey kidney (Vero) cells were both cultured in minimum essential medium (MEM; HyClone, South Logan, UT, USA) supplemented with 10% heat-inactivated fetal bovine serum (FBS; PAN, Adenbach, Freistaat Bayern, Germany) and 100,000 U/L penicillin plus 100,000 g/L streptomycin at 37 °C and 5% CO_2_. HeLa cells were grown in Dulbecco’s minimum essential medium (DMEM; HyClone, South Logan, UT, USA) supplemented with 10% heat-inactivated FBS and antibiotics (100,000 U/L penicillin and 100,000 g/L streptomycin) at 37 °C and 5% CO_2_. All cell lines were purchased from the China Center for Type Culture Collection (CCTCC, Wuhan, China).

RD cells were transfected with synthetic oligonucleotides or DNA plasmids using TurboFect transfection reagent (Thermo Scientific, Waltham, MA, USA) according to the manufacturer’s protocol. Synthetic oligonucleotides representing the hsa-let-7c-5p mimic, negative control miRNA mimic, hsa-let-7c-5p inhibitor and negative control miRNA inhibitor were purchased from Ribo Biotech (Guangzhou, China), and they were dissolved in RNase-free H_2_O at 20 μM for storage (at −80 °C).

A JNK inhibitor, SP600125, was purchased from Sigma-Aldrich (St. Louis, MO, USA) and dissolved in DMSO (Sigma, St. Louis, MO, USA) at 10 mM, and this mixture was added to fresh culture medium (supplemented with 1% heat-inactivated FBS and antibiotics) at a final concentration of 20 μM for 1 h prior to viral infection and kept in the medium throughout the experiment.

### EV71 infection

Cells were starved, infected with EV71 (BrCr strain, GenBank accession No. U22521) at a multiplicity of infection (MOI) of 5 and incubated for 1 h at 37 °C to allow for absorption of the virus. Then, the inoculum was removed, and incubation was continued in fresh culture medium supplemented with 1% heat-inactivated FBS and antibiotics at 37 °C and 5% CO_2_. Infected cells and culture supernatants were collected together at the time points post-infection indicated in the text and figure legends and clarified by centrifugation (2000*g*). Finally, virus titers were determined by plaque assays.

To generate UV-inactivated virus, 2-mL aliquots of the virus were dispersed in a 6-cm tissue culture dish and then exposed to UV under a compact UV lamp (UVP, Upland, CA) for 1 h on ice. The remaining titer of the inactivated virus was determined by plaque assay [56].

### Plasmid construction

To generate a psi-MAP4K4-3′UTR recombinant plasmid, a 927-nt partial fragment of the MAP4K4 3′UTR (GenBank accession No. NM_145686) was subcloned into a psiCHECK™-2 vector (Promega, Madison, WI, USA) using *Xho*I and *Not*I restriction sites. psi-MAP4K4-3′UTRmut was generated by overlapping PCR; this plasmid contained a mutated seed sequence, which was changed from CUACCUC to C*AU*C*GA*C. The restriction enzymes used in this experiment were purchased from Takara Bio (Dalian, China), and the LV-shMAP4K4 vector was obtained from Longqian Biotech (Shanghai, China). The primers used for plasmid construction are listed in Additional file 1: Table S1.

### RNA extraction and qRT-PCR

Total RNA was extracted from cell lines with TRIzol Reagent (Invitrogen, Carlsbad, CA, USA), and 2 μg isolated RNA was then reverse transcribed using a RevertAid First Strand cDNA Synthesis Kit (Thermo Scientific, Waltham, MA, USA). qRT-PCR was performed with SYBR Green Real-Time PCR Master Mix (Toyobo, Osaka, Japan) according to the manufacturer’s instructions. The PCR conditions for measuring mRNA and EV71 RNA levels were as follows: 95 °C for 3 min, followed by 40 cycles at 95 °C for 10 s, 60 °C for 15 s and 72 °C for 20 s. GAPDH was used as an internal control, and the PCR primers used have been previously described [57]. In addition, the PCR conditions for measuring hsa-let-7c-5p expression were as follows, with U6 snRNA used as an internal control: 95 °C for 20 s, followed by 40 cycles at 95 °C for 10 s, 60 °C for 20 s and 70 °C for 10 s. The primers used for reverse transcription and qRT-PCR of U6 snRNA have been previously described [58]. The sequence of the primer used for the reverse transcription of hsa-let-7c-5p was 5′-GTCGTATCCAGTGCAGGGTCCGAGGTATTCGCACTGGATACGACAACCAT-3′. The qRT-PCR primers used are listed in Additional file 1: Table S2. The data were analyzed using the 2^−△△ct^ method.

### Plaque assay

Vero cell monolayers were prepared by the addition of 0.5 mL cells to each well of 48-well plates at a concentration of 1 × 10^5^ cells per mL in MEM supplemented with 10% heat-inactivated FBS and antibiotics (Corning, NY, USA). The cells were incubated for 1 d before EV71 infection. Then, the medium was removed, and the cells were infected with 200 μL of a serial 10-fold-diluted viral suspension (with three replicates for each dilution). Next, the cells were incubated at 5% CO_2_ and 37 °C for 1 h, and the viral solution was aspirated. Subsequently, 500 μL of MEM supplemented with 1% heat-inactivated FBS and antibiotics plus 1% carboxymethyl cellulose was added to the wells. Then, the plates were incubated at 37 °C and 5% CO_2_ for 3 d, and the cells were fixed with methanol for 1 h at 4 °C and subsequently stained with crystal violet for 10 min. Plaques were counted manually.

### MTT assay

RD cells were seeded in 96-well plates (Corning, NY, USA) at 1 × 10^4^ cells per well. Then, the cells were transfected with the hsa-let-7c-5p mimic, miRNA inhibitor, LV-shMAP4K4 plasmid or corresponding negative control according to the manufacturer’s instructions. After 4 h of incubation, the medium was aspirated, and then fresh MEM supplemented with 1% heat-inactivated FBS and antibiotics was added. After 48 h of transfection, the cells were further infected with or without EV71 for 24 h, followed by incubation with 20 μL of 5 mg/mL MTT for 4 h. A volume of 150 μL DMSO was used to solubilize the formazan converted by MTT, and the plates were shaken for 10 min. Abosorbance was detected using a microplate reader (BioTek, VT, USA) at an OD of 490 nm.

### Luciferase assay

RD cells were grown in 24-well plates (Corning, NY, USA) to 70% confluency and then transfected with a synthetic oligonucleotide or DNA plasmid, as indicated in the text and figure legends. At specific time points post-transfection, the cells were harvested and assayed using a Dual-Luciferase Reporter Assay System (Promega, Madison, WI, USA) according to the manufacturer’s instructions.

### Western blotting analysis

RD cells were lysed on ice with RIPA buffer (Biosharp, China) containing phenylmethanesulfonyl fluoride (PMSF; Biosharp, China), and then the cell lysates were centrifuged at 10,000*g* for 5 min at 4 °C, and the pellets were discarded. The protein samples were separated in 8–12% polyacrylamide gels and then transferred to polyvinylidene difluoride (PVDF) membranes (Millipore, Hertfordshire, UK). The membranes were blocked with 5% non-fat milk powder or bovine serum albumin (BSA) in Tris-buffered saline containing 0.1% Tween 20 (TBST) at room temperature for 2 h and then incubated overnight at 4 °C with the following primary antibodies (except for a membrane incubated with a mouse polyclonal antibody to enterovirus 71 for 48 h): a mouse polyclonal antibody to enterovirus 71 (1 μg/mL; ab 169442, Abcam, Cambridge, MA, USA), goat polyclonal antibody to human HGK (1 μg/mL; AF6027, R&D, Minneapolis, MN, USA), rabbit monoclonal antibody to phospho-SAPK/JNK (diluted 1:1000; Thr183/Tyr185, #4688, CST, MA, USA), rabbit anti-SAPK/JNK polyclonal antibody (diluted 1:1000; #9252, CST, MA, USA), rabbit polyclonal antibodies to NF-κB and phospho-NF-κB (diluted 1:1000; CST, MA, USA), and GAPDH mouse monoclonal antibody (diluted 1:5000; PMK043C, PMK, Wuhan, China). Blots were washed and incubated for 1–2 h at room temperature with goat anti-rabbit, rabbit anti-mouse and anti-goat horseradish peroxidase-labeled antibodies (diluted 1:25,000; PMK). Protein band signals were detected by enhanced chemiluminescence (ECL; Thermo scientific, Waltham, MA, USA). Band densities were measured by densitometry and quantified using Image J software (version 1.46r, Bethesda, MD, USA).

### Statistical analysis

All experiments were performed in triplicate. The data are expressed as the mean ± standard derivation (SD) and were analyzed by analysis of variance (ANOVA) or Student’s *t* test. Significant differences were indicated by a *p* < 0.05.

## Additional file

**Additional file 1.** Additional tables and figures.

## Abbreviations

EV71: enterovirus 71
HFMD: hand, foot and mouse disease
miRNAs: microRNAs RD: rhabdomyosarcoma
MAP4K4: mitogen-activated protein kinase kinase kinase kinase 4
JNK: c-Jun NH2-terminal kinase
CPE: cytopathic effect
MOI: multiplicity of infection
MTT: 3-(4,5-dimethylthiazol-2-yl)-2,5-diphenyltetrazolium bromide
