# Supplementary material for: Hsa-let-7c-5p augments enterovirus 71 replication through viral subversion of cell signaling in rhabdomyosarcoma cells
Source: Cell Biosci. 2017 Jan 14;7:7. doi: 10.1186/s13578-017-0135-9 (PMC5237547; doi:10.1186/s13578-017-0135-9)
Supplement: Supplementary file 1 — Additional file 1. Additional tables and figures. [file 13578_2017_135_MOESM1_ESM.docx]

**Supplementary data**


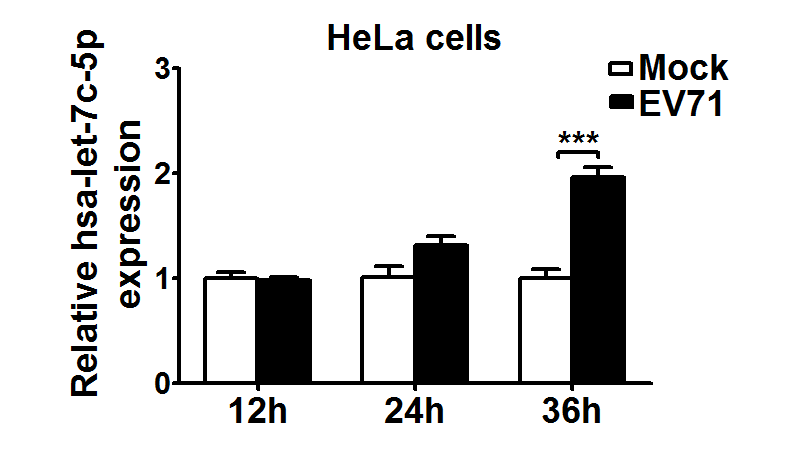


**Figure S1** Upregulation of hsa-let-7c-5p expression in EV71-infected HeLa cells. HeLa cells were infected with or without EV71 (MOI = 5) at the indicated time points. The expression of hsa-let-7c-5p was detected by stem-loop qRT-PCR and normalized to that of U6 snRNA. ***, *p* < 0.001.


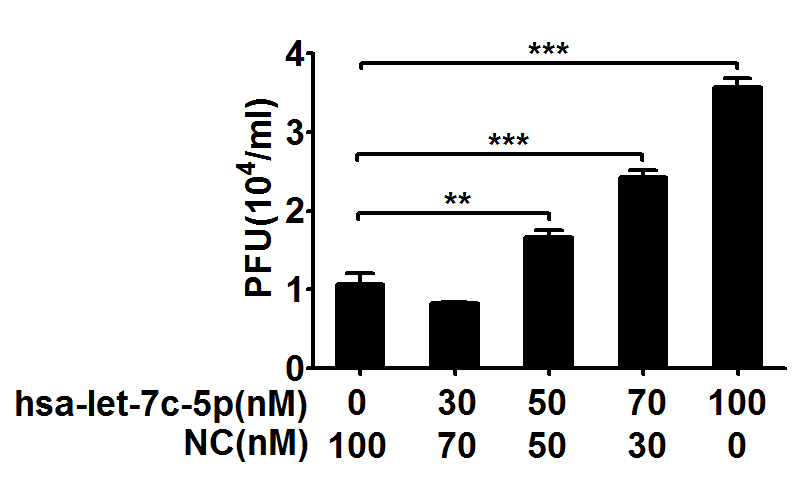


**Figure S2** Regulation of the EV71 titer by the hsa-let-7c-5p mimic at various concentrations. RD cells were co-transfected with the NC and hsa-let-7c-5p mimics at the indicated concentrations for 48 h, followed by EV71 infection (MOI = 5 ) for 24 h. Total virus was collected from infected cells and culture supernatants and used in plaque assays. The virus titers in the cultures are expressed as PFU per milliliter. **, *p* < 0.01; ***, *p* < 0.001.


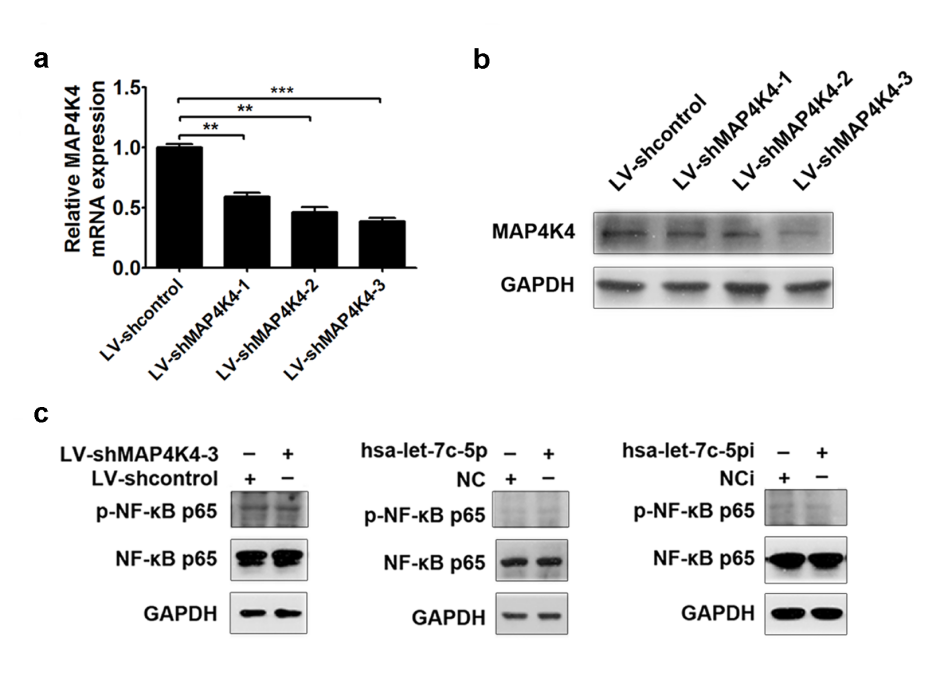


**Figure S3** MAP4K4 and hsa-let-7c-5p have no impact on NF-κB activation. (**a**) The mRNA and (**b**) protein levels of MAP4K4 targeted by MAP4K4 shRNAs were detected by qRT-PCR and Western blotting, respectively, following transfection of the cells with the shRNAs for 48 h. (**c**) Regulation of the NF-κB pathway by MAP4K4 and hsa-let-7c-5p. RD cells were transfected with the LV-shMAP4K4-3 vector, hsa-let-7c-5p mimic (100 nM), hsa-let-7c-5p inhibitor (150 nM) or corresponding control for 48 h. The phosphorylated and total protein levels of NF-κB were determined by Western blotting. **, *p* < 0.01; ***, *p* < 0.001.

**Table S1 Primers used for plasmids construction**

| **Primer names** | **Sequences** |
| --- | --- |
| psi-MAP4K4-3'UTR Forward | 5'-GCACTCGAGGATATGTGGACAAAGAAGAAG-3' |
| psi-MAP4K4-3'UTR Reverse | 5'-CCAGCGGCCGCATATTACACCTAAAACACA-3' |
| psi-MAP4K4-3'UTRmut Forward | 5'-TTTGCCATCGACATTGTTCTTAATGCATTG-3' |
| psi-MAP4K4-3'UTRmut Reverse | 5'-CAATGCATTAAGAACAATGTCGATGGCAAA-3' |
| LV-shMAP4K4-1 sense | 5'-CCGGGAGAAAGATGAAACTGAGTATCTCGAGA  TACTCAGTTTCATCTTTCTCTTTTTG-3' |
| LV-shMAP4K4-1 anti-sense | 5'-AATTCAAAAAGAGAAAGATGAAACTGAGTATC  TCGAGATACTCAGTTTCATCTTTCTC-3' |
| LV-shMAP4K4-2 sense | 5'-CCGGGCTTAAGGATCATATAGATCGCTCGAGC  GATCTA TATGATCCTTAAGCTTTTTG-3' |
| LV-shMAP4K4-2 anti-sense | 5'-AATTCAAAAAGCTTAAGGATCATATAGATCGCT  CGAGCGATCTATATGATCCTTAAGC-3' |
| LV-shMAP4K4-3 sense | 5'-CCGGGGATGAGACCAGAAGCCATAACTCGAGTT  ATGGCTTCTGGTCTCATCCTTTTTG-3' |
| LV-shMAP4K4-3 anti-sense | 5'-AATTCAAAAAGGATGAGACCAGAAGCCATAACT  CGAGTTATGGCTTCTGGTCTCATCC-3' |

**Table S2 Primers used for qRT-PCR**

| **Primer names** | **Sequences** |
| --- | --- |
| hsa-let-7c-5p Forward | 5'-GCGGCGTTGAGGTAGTAGGT-3' |
| hsa-let-7c-5p Reverse | 5'-GTGCAGGGTCCGAGGTATTC-3' |
| EV71-5'UTR sense | 5'-GCCCCTGAATGCGGCTAATC-3' |
| EV71-5'UTR anti-sense | 5'-CCAAAGTAGTCGGTTCCGCTGC-3' |
| MAP4K4 sense | 5'-GCACACTCCAGAAACACAAATC-3' |
| MAP4K4 anti-sense | 5'-CCTTATGGCTTCTGGTCTCATC-3' |
